# Let It Flow: Morpholino Knockdown in Zebrafish Embryos Reveals a Pro-Angiogenic Effect of the Metalloprotease Meprin α_2_

**DOI:** 10.1371/journal.pone.0008835

**Published:** 2010-01-21

**Authors:** André Schütte, Jana Hedrich, Walter Stöcker, Christoph Becker-Pauly

**Affiliations:** Institute of Zoology, Cell and Matrix Biology, Johannes Gutenberg-University, Mainz, Germany; University of Oldenburg, Germany

## Abstract

**Background:**

Meprin metalloproteases are thought to be involved in basic physiological functions such as cell proliferation and tissue differentiation. However, the specific functions of these enzymes are still ambiguous, although a variety of growth factors and structural proteins have been identified as meprin substrates. The discovery of meprins α_1_, α_2_ and β in teleost fish provided the basis for uncovering their physiological functions by gene silencing *in vivo*.

**Methodology/Principal Findings:**

A Morpholino knockdown in zebrafish embryos targeting meprin α_1_ and β mRNA caused defects in general tissue differentiation. But meprin α_2_ morphants were affected more specifically and showed severe failures in the formation of the vascular system provoking the hypothesis of a pro-angiogenic effect. The blood circulation was largely diminished resulting in erythrocyte accumulation. These phenotypes mimic a previously described VEGF-A morphant, revealing a possible role of meprin α in VEGF-A activation. Indeed, human recombinant meprin α processed the vascular endothelial growth factor-A (VEGF-A) specifically, revealing the same cleavage products detectable for VEGF from zebrafish whole lysate.

**Conclusions/Significance:**

Our results demonstrate that meprin metalloproteases are important for cell differentiation and proliferation already during embryogenesis, predominantly by the activation of growth factors. Thus, we conclude that meprins play a significant role in VEGF-A processing, subsequently regulating angiogenesis. Therefore, meprin α might be a new therapeutic target in cardiovascular diseases or in tumor growth inhibition.

## Introduction

Meprin α and β exhibit unique features within the astacin family of zinc endopeptidases and the metzincin superfamily [1], [2]. So far they could only be identified in vertebrates like fish, platypus, rodents and humans [3], [4], [5], [6], [7]. Striking are the molecular properties, revealing meprin α as the largest secreted protease known so far, due to oligomerisation up to 6 megadalton units [8], [9]. Moreover, meprin β is the only astacin that stays predominantly membrane bound [10].

Originally, meprin expression has been observed on the apical side of epithelial brush borders in kidney proximal tubules and small intestine [5], [6]. In the meantime, various other tissues have been found to express meprins differentially [11], [12]. Meprins are secreted as zymogens, which are activated by proteolytic removal of amino terminal propeptides. Several ways for activation have been unraveled, depending on the expressing tissue. In the gut, both human enzymes are converted to their mature forms by trypsin [8]. Outside the intestine, there is selective activation of meprin α by plasmin [13], and of meprin β by tissue kallikrein-related peptidase 4 (KLK4) [11], respectively. Upon secretion into the extracellular matrix (ECM), meprins are able to cleave a number of ECM proteins like laminin, fibronectin, collagen IV and nidogen, peptide hormones like bradykinin [10], [14], as well as cytokines and growth factors like TGF-α, interleukin-1β and interleukin-18 [12], [14], [15], [16].

The observed activation of interleukins by meprins and their expression in leukocytes of the intestinal *lamina propria* indicate a function in the immune response. This is supported by reports on a role for meprins in inflammatory bowel disease or Crohn's disease [17], [18], [19]. Furthermore, meprin α is expressed in certain tumors such as colorectal cancer and, hence, might play a role in tumor cell migration and invasion, and cancer progression [13], [20]. Other sites of expression are human keratinocytes, where meprin α and β are found in the s*tratum basale* and in the s*tratum granulosum*, respectively [11].

In zebrafish, three homologous meprins, two meprin α variants and one β, are expressed in a broad array of tissues. Besides intestine and skin, the proteases could be found in kidney, head kidney, brain, gills, heart and liver [3]. Thus, the zebrafish appeared as a well suited model for studying the physiological function of meprins *in vivo*, which is made feasible by using morpholinos for knocking down meprin genes in zebrafish embryos.

## Results and Discussion

**Figure 1:**
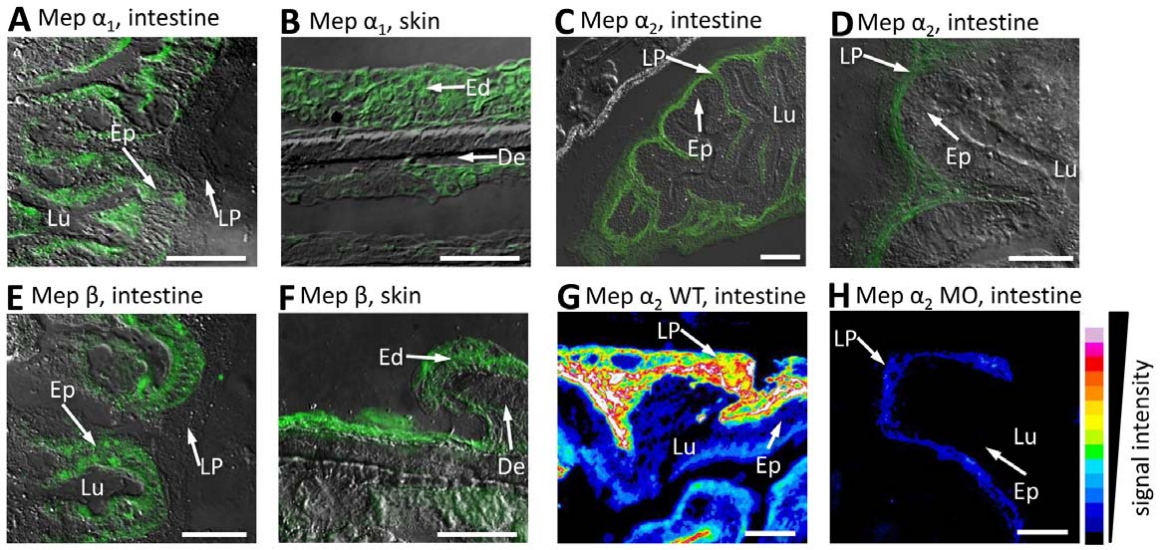
Distribution of meprin α_1_, α_2_ and β in zebrafish tissues. Immunofluorescence microscopy of cryosections from whole mount zebrafish, using specific peptide antibodies, revealed meprin α_1_ in the brush border cells of intestinal epithelia (Ep) (A) and epidermis (B), whereas meprin α_2_ was observed in the *lamina propria mucosae* (LP) only (C, D). Additionally, meprin β signals could be detected in the zebrafish intestine (Ep) and epidermis (Ed) (E, F respectively). To verify the efficiency of meprin knockdowns due to morpholino injection, we compared the fluorescence signal intensity in cryosections of wild type (G) and meprin α_2_ deficient embryos (H). Evidently, the expression of meprin α_2_ in the *lamina propria (LP)* of the intestine is largely decreased (G, H). Similar analyses of the meprin β morphant were not possible, due to the lethality within 24 hpf. (Ep: epithelium; Lu: lumen; LP: lamina propria; Ed: epidermis; De: dermis; scale bars: 25 µm; signal intensity was calculated with ImageJ V.1.41o).

Peptide antibodies were generated against each of the three meprin variants and used to examine cryosections of 16 weeks old zebrafish by immunofluorescence microscopy, which revealed the intestine as the main expression site for all meprins, and the epidermis for meprin α_1_ and β. Within the gut, meprin α_1_ as well as β could be observed only in the brush border cells of the intestinal epithelia (Figure 1A, E), whereas meprin α_2_ signals were detected in the *lamina propria mucosae* (Figure 1C). The distribution of fluorescence signals implies that meprin α_2_ expression could occur in close proximity to endothelial cells (Figure 1D). The distinct expression pattern of all three proteases indicates different functions *in vivo*. Recently it was shown that meprins are involved in certain intestinal pathologies like inflammatory bowel disease (IBD) or ulcerative colitis [21]. Meprin β^−/−^ mice exhibit a diminished activation of pro-interleukin-18 (IL-18), which plays a key role in IBD [16]. Similar to IL-18, also IL-1β is processed by meprin β to its mature form [15].

Expression of meprin α_1_ and β in zebrafish epidermis (Figure 1B, F) correlates to the situation in human skin. We could show that meprin α and β are expressed in separate cell layers of human epidermis [11]. This is reflected by diverse effects of the recombinant enzymes on cultured keratinocytes (HaCaT). Here, meprin β induced a dramatic change in cell morphology and reduced the cell number, whereas meprin α seem to play a role in basal keratinocyte proliferation.

**Figure 2:**
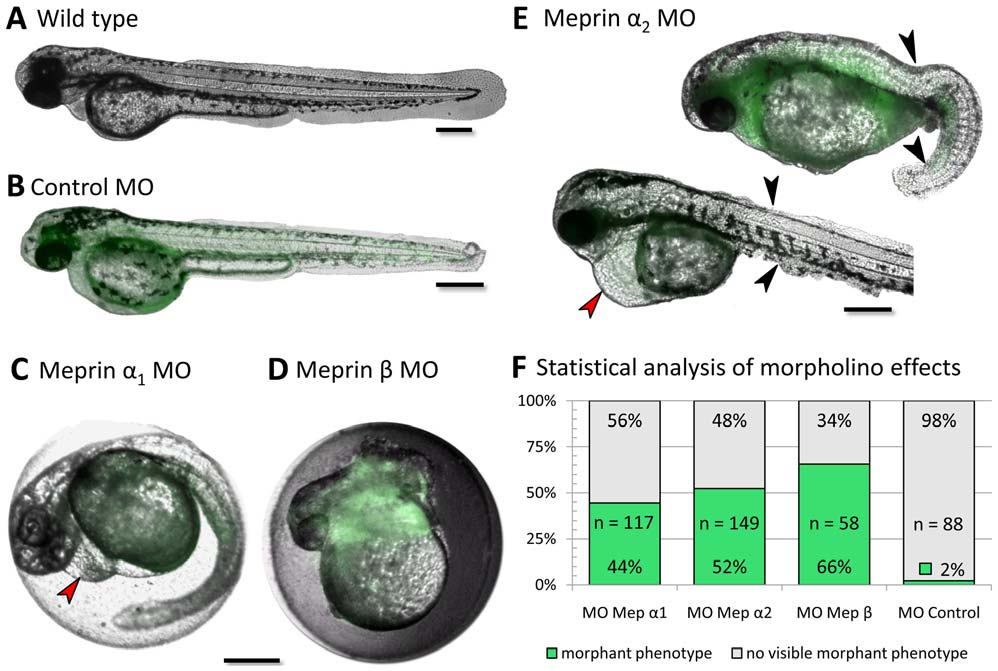
Morpholino knockdown in zebrafish embryos exhibit severe phenotypes of meprin α_1_, α_2_ and β (C–E). Wild type (A, 60 hpf) and control fish (B, 42 hpf) did not show any defects in embryonic morphology and development. In meprin α_1_ morphants (C, 32 hpf), only slight defects like the dilation of pericard (red arrow) were visible, whereas meprin β morphants (D, 22 hpf) showed striking tissue disorganization in trunk and tail, leading to early death within 24 hpf. Embryos injected with morpholinos against meprin α_2_ (E, 42 hpf), likewise exhibited dilated pericards (red arrow), but also showed severe epidermal abnormalities in trunk and tail (black arrows). Statistical analyses visualize the frequency of morphant phenotypes (F). ‘n’ describes the number of injected embryos. Morpholinos were tagged with carboxyfluorescein (green fluorescence). (Scale bars: 250 µm).

All three meprins (α_1_, α_2_ and β) were identified by RT-PCR in developing zebrafish embryos starting at 4 hours post fertilization (hpf; data not shown). Thus, they are expressed at the end of the blastula stage, which is typical for zygotic genes activated during midblastula transition [22], [23]. This suggests important roles for meprins in tissue formation and assembly in early embryonic development. To elucidate the functions of the three zebrafish meprins *in vivo*, we created knockdown embryos using morpholinos targeting each protease specifically [24]. As controls, “standard morpholino oligomers” were injected, not targeting any gene in zebrafish (Figure 2A). Consequently, 98% of the injected control larvae did not show any morphant phenotype and the remaining 2% correspond to natural mortality, likewise observed for untreated embryos (Figure 2B, F). To prove the successful knockdown we compared the intensity of fluorescence signals for meprin α_2_ in cryosections of injected and wild type animals (see Figure 1G, H).

Obviously, the expression of meprin α_2_ in the *lamina propria* of zebrafish intestine was significantly decreased in morphant animals (Figure 1H). Meprin α_1_ knockdown animals showed relatively mild, but clearly distinct alterations in comparison to wild type animals (Figure 2C, A respectively). 44% of the injected embryos revealed a dilated pericardium or a distorted trunk and tail tissue, probably due to disorders in cell differentiation (Figure 2F). By contrast, meprin β knockdown animals exhibited strikingly abnormal disorders of the whole trunk and tail in early development (Figure 2D). Overall, the tissues seemed to be unstructured lacking any normal cell differentiation. The morphant embryos are viable in the beginning, but die within 24 hours post injection. This phenotype reveals very distinct and fundamental functions for meprin β in the differentiation of cells during embryonic development.

**Figure 3:**
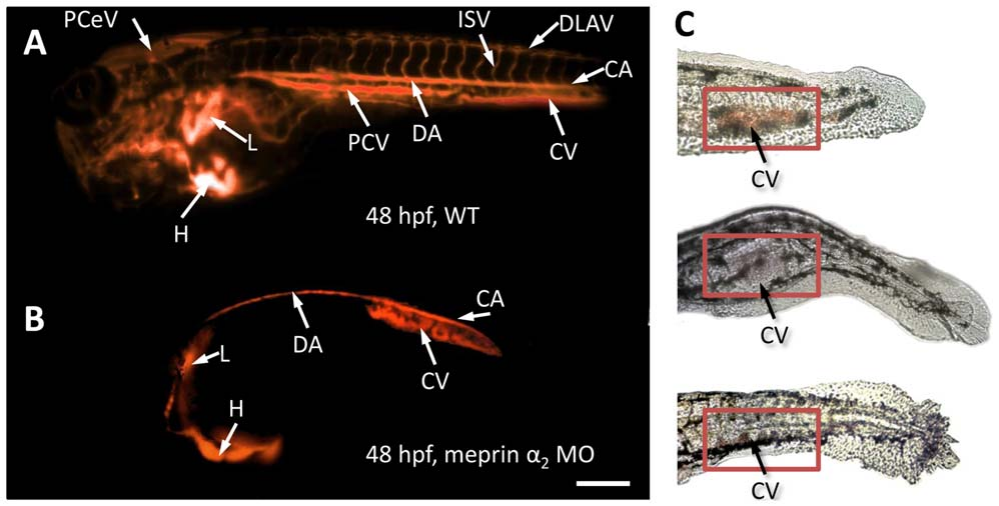
The vascular system of meprin α_2_ knockdown embryos exhibits dramatic defects (B, C). Microangiography (with TRITC-Dextran) revealed a drastically reduced vascular system without any intersegmental blood vessels (B) in meprin α_2_ morphants, compared to the non-injected wild type zebrafish (A). Additionally, erythrocytes accumulated in the ventral caudal tail region (C), possibly as a consequence of this sprouting failure. (PCeV: Posterior cerebral vein; ISV: intersegmental vessels; DLAV: dorsal longitudinal anastomotic vessel; CA: caudal artery; DA: dorsal aorta CV: caudal vein; PCV: posterior cardinal vein; L: liver; H: heart) (Scale bar: 250 µm).

**Figure 4:**
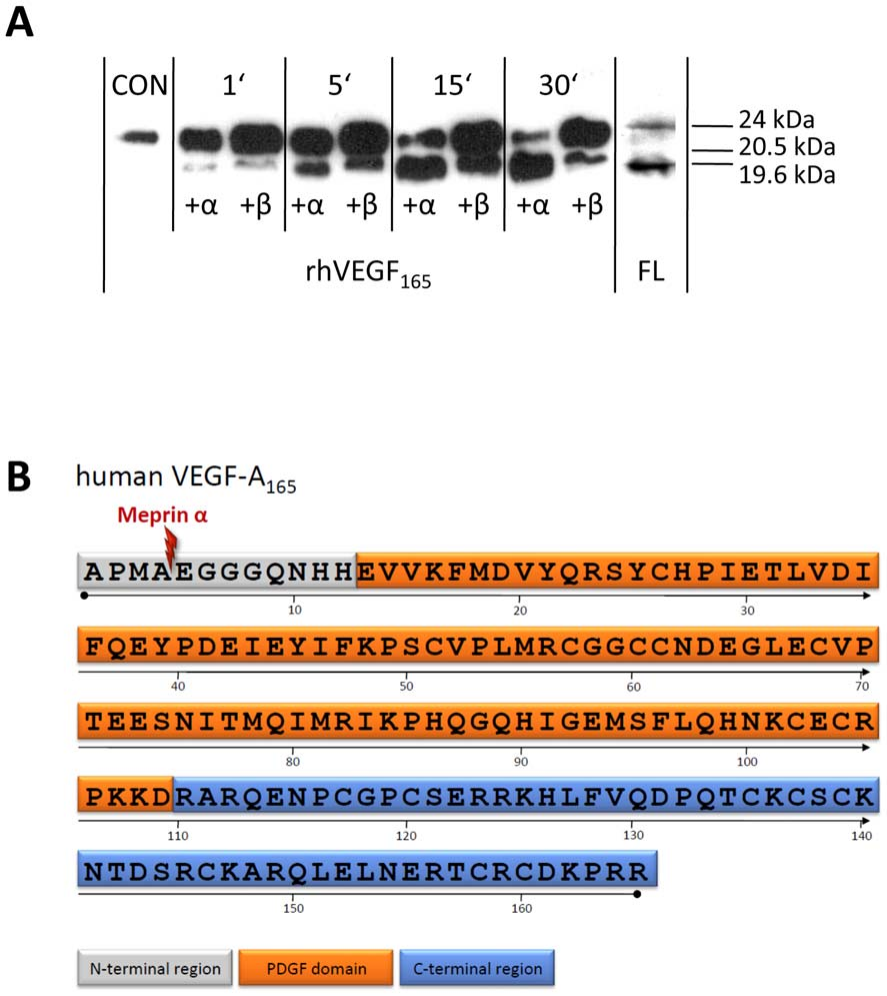
Human meprin α and β are capable of processing VEGF-A specifically. (A) The cleavage of recombinant human VEGF-A_165_ (CON, untreated, 24 kDa) by recombinant meprin α and β (each 85 nM) for 1 to 30 min resulted in two fragments of different molecular weight (19.6 kDa in case of meprin α, 20.5 kDa by meprin β), visualized by western blot analysis. In wild type zebrafish whole lysates (FL), VEGF-A could be detected using specific antibodies indicating a fragment similar to that produced by meprin processing. (B) Domain structure of human VEGF-A_165_ (P15692-4). The lightning indicates the cleavage site between Ala4 and Glu5, identified after incubation with recombinant human meprin α. PDGF (platelet-derived growth factor). In addition, the N-terminal cleavage site in human VEGF-A_165_ between Ala30 and Glu31 due to recombinant human meprin α activity could be identified by N-terminal sequencing.

In the case of meprin α_2_ knockdowns the epidermal cell layers seem to be widely disorganized in the trunk and especially in the tail region (Figure 2E). But the most informative phenotype became visible in meprin α_2_ morphants beyond the age of 48 hpf. These embryos exhibited a dramatically degenerated vascular system and the blood circulation was largely diminished or even completely interrupted (Figure 3B). Consequently, red blood cells accumulated ventrally in the caudal region of a considerable number of phenotypes (see Figure 3C). To visualize the blood vessels in living embryos, tetramethyl rhodamine isothiocyanate-Dextran (TRITC-Dextran) was injected at the age of 48 hpf for microangiography [25]. This method uncovered the almost complete absence of intersegmental vessels (ISV) (Figure 3B), which normally begin to sprout at the 26-somite stage (21 hpf) [26] in wild type embryos (Figure 3A). The only prominent vessel was the large dorsal aorta (DA) extending ventrally from the heart to the tail vessels (Figure 3B; supporting movie file Video S1). These phenotypes mimic even in detail previously described VEGF-A (vascular endothelial growth factor A) morphants, regarding the reduced vascular system and erythrocyte accumulation [27]. In corresponding morphants, the loss of the VEGF receptor flk-1 (VEGFR-2) resulted in the absence of angiogenic sprouting of all blood vessels as a consequence of disorganized endothelia [28]. Based on the morphant phenotypes observed here, we argued that meprin α_2_ might be involved in angiogenic blood vessel formation by processing VEGF-A. To test this hypothesis, we incubated recombinant human meprin α and β with recombinant human VEGF-A_165_, which is the predominant isoform in humans. By western blot analysis using a specific VEGF-A antibody, we were able to demonstrate that both human meprin α and β cleaved VEGF-A by limited proteolysis. This yielded in two distinct fragments of 19 kDa (meprin α) and 20 kDa (meprin β), derived from the 24 kDa unprocessed VEGF-A_165_ monomer (Figure 4A). Moreover, by western blotting we detected zebrafish VEGF in cell lysates of wildtype fish, displaying the same cleavage pattern in accordance to the cleavage of human VEGF-A by meprin α (Figure 4A). By N-terminal sequencing, we were able to identify the cleavage site in VEGF-A_165_ incubated with meprin α, between Ala4 and Glu5 (Figure 4B). Since this cleavage is not decisive for the different molecular weights of the emerging fragments, we propose, that VEGF-A is cleaved additionally within the C-terminal region. However, no distinctive bands could be observed corresponding to C-terminal fragments, probably due to multiple processing events (data not shown).

Hence, meprin metalloproteases might trigger angiogenesis in two possible ways. On the one hand, unique N-terminal processing of the growth factor could increase its potential to enhance endothelial cell proliferation. On the other hand, removal of the inhibitory C-terminal region from the anti-angiogenic factor VEGF-A_xxx_b would likewise cause a pro-angiogenic effect [29].

The disturbed organization of the epidermal cells and the deformation of tail and trunk as seen in meprin α and β morphants could be due to the proteolytic activity of meprins on cytokines like VEGF. This assumption is supported by the co-localization of meprins and VEGF in human keratinocytes. Here, VEGF plays an important role in permeability barrier homeostasis and dermal angiogenesis [30]. It has been shown previously that meprins cleave various other cytokines, growth factors and peptides, which take part in different situations like cell migration or tissue formation. For instance, TGF-α and IL-8 are processed and thereby activated by meprin α during inflammatory disease in human lung [12].

In summary, we could demonstrate by *in vivo* knockdown studies that meprins have fundamental physiological effects in the early embryonic development of zebrafish. The data shows that meprin metalloproteases are involved in general tissue differentiation. Moreover, we conclude that meprin α_2_ is required to process VEGF-A, thereby triggering angiogenesis in the zebrafish.

## Methods

### Fish Maintenance

Zebrafish (*Danio rerio*) were bred and kept under constant conditions at a temperature of 28°C and a schedule of 14 h light and 10 h darkness. From embryonic stadium, fish were fed daily with dry food and weekly with living food (*Artemia salina*). Embryos were staged according to morphological criteria [23].

### Morpholino Sequences

Antisense-morpholino phosphorodiamidate oligonucleotides were designed against following sequences (GeneTools, Philomath, USA).

meprin α_1_: 5′- AGA TGA GCA GTC TCT GTA AAA GCA T -3′

meprin α_2_: 5′- GGC TGA TTC TCC ACA TGG AGT CCA T -3′

meprin β: 5′- AGA GAT AGG AAC AAG CAG ACG CCA T -3′.

Each oligo was tagged with 3′ fluorescein to visualize the distribution in the injected cells. As control, a standard morpholino oligo targeted against a mutation in the human beta-globin pre-mRNA was used (5′-CCT CTT ACC TCA GTT ACA ATT TAT A-3′).

### Morpholino Microinjection

Morpholino oligonucleotides were diluted with Danieu buffer to a concentration of 0.3 mM. (Danieu buffer: 58 mM NaCl, 0.7 mM KCl, 0.4 mM MgSO_4_, 0.6 mM Ca(NO_3_)_2_, 5 mM HEPES, pH 7.6). Zebrafish eggs were fixed in appropriate furrows on a 1.5% agarose plate. 4 ng of Morpholinos were then injected into the one- or two-cell stages by using a micromanipulator (Märzhäuser, Wetzlar, Germany) and microinjector (Transjector 5246, Eppendorf, Hamburg, Germany). Injected embryos were raised in 96-well-plates with embryo medium (5 mM NaCl, 0.17 mM KCl, 0.33 mM CaCl_2_, 0.33 mM MgSO_4_). To avoid fungal growth; 0.1% methylene blue was added to the medium.

### Microangiography

Embryos of the age of 2 days post fertilization (dpf) were anesthetized using tricaine (MS-222, 40 µg/ml, Sigma-Aldrich, Deisenhofen, Germany). Then, TRITC (Tetramethyl rhodamine isothiocyanate, 20 mg/ml; Sigma-Aldrich, Deisenhofen, Germany) was injected into the circular system through the posterior cardinal vein using the microinjection system described above [25]. The injected embryos were then examined by fluorescence microscopy, using a DM IRBE microscope (Leica, Wetzlar, Germany).

### Tissue Lysis and Western Blot Analysis

Homogenized adult fish were incubated in lysis buffer (137 mM NaCl, 2.7 mM KCl, 9.2 mM Na_2_HPO_4_, 1.8 mM KH_2_PO_4_, 1% Triton X-100, pH 7.4) overnight at 4°C. After separation from cell debris by centrifugation at 13.200×g for 5 min., the lysate was concentrated using Amicon centrifugal filter units with an exclusion size of 50 kDa (Millipore, Eschborn, Germany). For immunoblot analysis proteins were subjected to 14% SDS-PAGE under reducing conditions and afterwards transferred onto a polyvinylidene fluoride (PVDF)-membrane (Immobilon P, Millipore, Eschborn, Germany) by electro blotting (80 mA, 75 min). For blocking, the membrane was saturated with 3% bovine serum albumin (BSA) for 1 h at room temperature (RT), incubated with the primary monoclonal anti-zebrafish VEGF-A antibody (1∶200; R&D Systems, Wiesbaden, Germany) for 1 h and afterwards with horseradish peroxidase-conjugated anti-mouse IgG (1∶6250) for 45 min at room temperature. Between all these steps, the membrane was washed with TBS-T (20 mM Tris/HCl, 500 mM NaCl, 0.05% Tween20, 0.2% Triton-X-100) and TBS (20 mM Tris, 137 mM NaCl). Detection was performed using Rotilumin (Roth, Karlsruhe, Germany) following the manufacturer's instructions using X-ray film (Hyperfilm ECL, Amersham Pharmacia Biotech, Freiburg, Germany). MagicMark XP (Invitrogen, Karlsruhe, Germany) was used as a molecular weight marker.

### VEGF-A Substrate Assay

500 ng of recombinant human VEGF-A_165_ (Immunotools, Friesoythe, Germany) was incubated with 85 nM meprin α or meprin β, respectively, for different times (1, 5, 15 and 30 minutes) at 37°C. By western blotting, the resulting fragments were identified using an anti-human VEGF-A antibody (1∶200, VEGF(C-1) sc7269, Santa Cruz Biotechnology, Santa Cruz, USA). Recombinant human meprin α and β were expressed, purified and activated as described before [8], [11]. For N-terminal sequencing, proteins were blotted onto PVDF membranes, stained with Coomassie Brilliant Blue and sequenced at the protein micro-sequencing center of the Institut Fédératif de Recherche (IFR) 128 (Lyon, France). In zebrafish whole lysate (from adult fish), VEGF was detected by western blotting using a monoclonal anti-zebrafish VEGF antibody (R&D Systems, Wiesbaden, Germany).

### Immunofluorescence Analysis

Cryosections of unfixed 4 weeks old zebrafish were generated with the cryostat HM 560 (Microm, Walldorf, Germany) and incubated with 5% goat serum in phosphate buffered saline (PBS: 137 mM NaCl, 2.7 mM KCl, 9.2 mM Na_2_HPO_4_, 1.8 mM KH_2_PO_4_, pH 7.4) to block non-specific binding. Afterwards the samples were incubated for 2 h at 4°C with polyclonal anti-zebrafish meprin antibodies (1∶200 in 0.5% goat serum/PBS). The polyclonal antisera from rabbit and guinea pig were generated against the following peptides. IgG fractions were purified by a sepharose-6B-column (Pineda, Berlin, Germany):

meprin α_1_: NH_2_-CTLDPSDGFWRGPSK-CONH_2_

meprin α_2_: NH_2_-CHDAKVQSERFYNSEGYAY-CONH_2_

meprin β: NH_2_-CVREYTAENPKGDLRL-CONH_2_

After removal of unbound primary antibody by washing with PBS, the samples were incubated with Alexa 568 goat anti-rabbit IgG or Alexa 488 goat anti-guinea pig IgG fluorescent antibody, respectively (1∶400 in 0.5% goat serum/PBS; Invitrogen, Karlsruhe, Germany) for 90 min. Moreover, 4.6-Diamidino-2-phenylindol (DAPI) was added to label the nuclei. Immunofluorescence detection was carried out using a DM IRBE microscope (Leica, Wetzlar, Germany) with fluorescence facility. The fluorescence intensity was compared on cryosections from intestine of wild type zebrafish and meprin α_2_ morphants. The software ImageJ (U. S. National Institutes of Health, Bethesda, USA) was used to measure and visualize the grade of intensity.

### Accession Numbers

Proteins described in this work are deposited in the uniprot/TrEMBL database: Q5RHM1 (meprin α_1_), B3DKP9 (meprin α_2_), Q08CC4 (meprin β), Q16819 (human meprin α), Q16820 (human meprin β), P15692 (human VEGF-A).

## Supporting Information

Video S1 The movie shows the phenotype of the living meprin α_2_ knockdown zebrafish. First scene displays the embryo in bright field microscopy, with the visible heart beating. This is followed by fluorescence microscopy, revealing a degenerated vascular system compared to the wildtype fish, visualized by microangiography.
(3.15 MB MOV)

## References

[1] Bond JS, Beynon RJ (1995). The astacin family of metalloendopeptidases.. Protein science: a publication of the Protein Society.

[2] Stöcker W, Grams F, Baumann U, Reinemer P, Gomis-Rüth FX (1995). The metzincins–topological and sequential relations between the astacins, adamalysins, serralysins, and matrixins (collagenases) define a superfamily of zinc-peptidases.. Protein science: a publication of the Protein Society.

[3] Schütte A, Lottaz D, Sterchi EE, Stöcker W, Becker-Pauly C (2007). Two alpha subunits and one beta subunit of meprin zinc-endopeptidases are differentially expressed in the zebrafish Danio rerio.. Biological chemistry.

[4] Warren WC, Hillier LW, Marshall Graves JA, Birney E, Ponting CP (2008). Genome analysis of the platypus reveals unique signatures of evolution.. Nature.

[5] Beynon RJ, Shannon JD, Bond JS (1981). Purification and characterization of a metallo-endoproteinase from mouse kidney.. The Biochemical journal.

[6] Sterchi EE, Green JR, Lentze MJ (1982). Non-pancreatic hydrolysis of N-benzoyl-l-tyrosyl-p-aminobenzoic acid (PABA-peptide) in the human small intestine.. Clinical science (London, England: 1979).

[7] Oneda B, Lods N, Lottaz D, Becker-Pauly C, Stöcker W (2008). Metalloprotease meprin beta in rat kidney: glomerular localization and differential expression in glomerulonephritis.. PLoS ONE.

[8] Becker C, Kruse M-N, Slotty KA, Köhler D, Harris JR (2003). Differences in the activation mechanism between the alpha and beta subunits of human meprin.. Biological chemistry.

[9] Ishmael FT, Norcum MT, Benkovic SJ, Bond JS (2001). Multimeric structure of the secreted meprin A metalloproteinase and characterization of the functional protomer.. The Journal of biological chemistry.

[10] Bertenshaw GP, Norcum MT, Bond JS (2003). Structure of homo- and hetero-oligomeric meprin metalloproteases. Dimers, tetramers, and high molecular mass multimers.. The Journal of biological chemistry.

[11] Becker-Pauly C, Höwel M, Walker T, Vlad A, Aufenvenne K (2007). The alpha and beta subunits of the metalloprotease meprin are expressed in separate layers of human epidermis, revealing different functions in keratinocyte proliferation and differentiation.. The Journal of investigative dermatology.

[12] Bergin DA, Greene CM, Sterchi EE, Kenna C, Geraghty P (2008). Activation of EGFR by a novel metalloprotease pathway.. The Journal of biological chemistry.

[13] Rösmann S, Hahn D, Lottaz D, Kruse M-N, Stöcker W (2002). Activation of human meprin-alpha in a cell culture model of colorectal cancer is triggered by the plasminogen-activating system.. The Journal of biological chemistry.

[14] Kounnas MZ, Wolz RL, Gorbea CM, Bond JS (1991). Meprin-A and -B. Cell surface endopeptidases of the mouse kidney.. The Journal of biological chemistry.

[15] Herzog C, Kaushal GP, Haun RS (2005). Generation of biologically active interleukin-1beta by meprin B.. Cytokine.

[16] Banerjee S, Bond JS (2008). Prointerleukin-18 is activated by meprin beta in vitro and in vivo in intestinal inflammation.. The Journal of biological chemistry.

[17] Lottaz D, Hahn D, Müller S, Müller C, Sterchi EE (1999). Secretion of human meprin from intestinal epithelial cells depends on differential expression of the alpha and beta subunits.. European journal of biochemistry/FEBS.

[18] Lottaz D, Buri C, Monteleone G, Rösmann S, Macdonald TT (2007). Compartmentalised expression of meprin in small intestinal mucosa: enhanced expression in lamina propria in coeliac disease.. Biological chemistry.

[19] Crisman JM, Zhang B, Norman LP, Bond JS (2004). Deletion of the mouse meprin beta metalloprotease gene diminishes the ability of leukocytes to disseminate through extracellular matrix.. J Immunol.

[20] Matters GL, Manni A, Bond JS (2005). Inhibitors of polyamine biosynthesis decrease the expression of the metalloproteases meprin alpha and MMP-7 in hormone-independent human breast cancer cells.. Clinical & experimental metastasis.

[21] Banerjee S, Oneda B, Yap LM, Jewell DP, Matters GL (2009). MEP1A allele for meprin A metalloprotease is a susceptibility gene for inflammatory bowel disease.. Mucosal immunology.

[22] Kane DA, Kimmel CB (1993). The zebrafish midblastula transition.. Development (Cambridge, England).

[23] Kimmel CB, Ballard WW, Kimmel SR, Ullmann B, Schilling TF (1995). Stages of embryonic development of the zebrafish.. Developmental dynamics: an official publication of the American Association of Anatomists.

[24] Nasevicius A, Ekker SC (2000). Effective targeted gene ‘knockdown’ in zebrafish.. Nature genetics.

[25] Weinstein BM, Stemple DL, Driever W, Fishman MC (1995). Gridlock, a localized heritable vascular patterning defect in the zebrafish.. Nature medicine.

[26] Fouquet B, Weinstein BM, Serluca FC, Fishman MC (1997). Vessel patterning in the embryo of the zebrafish: guidance by notochord.. Developmental biology.

[27] Nasevicius A, Larson J, Ekker SC (2000). Distinct requirements for zebrafish angiogenesis revealed by a VEGF-A morphant.. Yeast (Chichester, England).

[28] Habeck H, Odenthal J, Walderich B, Maischein H, Schulte-Merker S (2002). Analysis of a zebrafish VEGF receptor mutant reveals specific disruption of angiogenesis.. Current biology: CB.

[29] Harper SJ, Bates DO (2008). VEGF-A splicing: the key to anti-angiogenic therapeutics?. Nature reviews Cancer.

[30] Elias PM, Arbiser J, Brown BE, Rossiter H, Man M-Q (2008). Epidermal vascular endothelial growth factor production is required for permeability barrier homeostasis, dermal angiogenesis, and the development of epidermal hyperplasia: implications for the pathogenesis of psoriasis.. The American journal of pathology.

